# Beverages Sales in Mexico before and after Implementation of a Sugar Sweetened Beverage Tax

**DOI:** 10.1371/journal.pone.0163463

**Published:** 2016-09-26

**Authors:** M. A Colchero, Carlos Manuel Guerrero-López, Mariana Molina, Juan Angel Rivera

**Affiliations:** 1 Center for Health Systems Research, Instituto Nacional de Salud Pública, Cuernavaca, Mexico; 2 Center for Research on Nutrition and Health, Instituto Nacional de Salud Pública, Cuernavaca, Mexico; Mexican Social Security Institute, MEXICO

## Abstract

**Objective:**

To estimate changes in sales of sugar sweetened beverages (SSB) and plain water after a 1 peso per liter excise SSB tax was implemented in Mexico in January 2014.

**Material and Methods:**

We used sales data from the Monthly Surveys of the Manufacturing Industry from January 2007 to December 2015. We estimated Ordinary Least Squares models to assess changes in per capita sales of SSB and plain water adjusting for seasonality and the global indicator of economic activity.

**Results:**

We found a decrease of 7.3% in per capita sales of SSB and an increase of 5.2% of per capita sales of plain water in 2014–2015 compared to the pre-tax period (2007–2013).

**Conclusions:**

Adjusting for variables that change over time and that are associated with the demand for SSB, we found the tax was associated with a reduction in per capita sales of SSB. The effectiveness of the tax should be evaluated in the medium and long term.

## Introduction

In Mexico, more than two thirds of the adult population and more than one third of children and adolescents are overweight or obese [1–3]. Although obesity is a multi-causal disease, evidence shows that consumption of sugar-sweetened beverages (SSB) is associated with weight gain, diabetes and other chronic diseases [4–9]. SSB consumption in Mexico is high as it represents on average 9.8% of total energy intake [10], higher than the recommended 3% [11].

In this context, the government implemented an excise tax of 1 Mexican peso per liter (about 5.5 US cents) to all non-alcoholic beverages with added sugar starting in January 2014. The tax applies to sodas, flavored waters, teas, sweetened dairies, energy drinks with added sugar and excludes beverages with artificial sweeteners and 100% juices. The implementation of the tax led to an 11% price increase in taxed carbonated sweetened beverages (sodas) and a slightly smaller increase in taxed non carbonated sweetened beverages (flavored water, juices, energy drinks) [12]. Price increases were different because non-carbonated beverages are more expensive and more price elastic compared to carbonated beverages [12,13]. Using data on purchases of beverages (both taxed and untaxed) from a panel of households in urban areas we showed an average 6% decline in purchases of taxed beverages and a 4% increase in untaxed beverages in 2014 [14].

Since analyses and publishing of results from consumer panel data require a substantial amount of time, aggregate sales data have been used in Mexico as a faster means to judge the effects of the tax during 2015, the second year of its enforcement. Various agencies and columnists in Mexico have concluded that the Mexican tax on sugar-sweetened beverages (SSB) did not reduce purchases of these products in 2015, as it did in 2014. This conclusion is based on comparisons of raw (unadjusted) aggregate sales data. However, these simple analyses do not consider factors other than the tax, which influence purchases and consumption of these beverages and that, may change from the periods before and after the tax [15]. Therefore, the objective of this paper was to compare changes in sales of sugar sweetened beverages and plain water before (2007–2013) and after the tax (2014–2015) using SSB sales both unadjusted and adjusted for factors which may influence purchases. To the best of our knowledge, no other study has been published documenting changes in consumption using sales data and with a longer post and pre tax period.

## Methods

### Source of data

We used sales data from the Monthly Surveys of the Manufacturing Industry (EMIM, for its acronym in Spanish) from January 2007 to December 2015 [16]. In this survey, the unit of observation is the manufacturing establishment, defined as an economic unit that has a fixed location and combines resources to transform or modify inputs into new products. EMIM has a deterministic sampling design: producers are selected from the economic census and the sample size is reached until the number of firms equals the expected coverage for each economic activity. Firms are selected from a pool ordered from the largest to the smallest. For food and beverages, coverage is defined as 80% of total income and 80% of employees.

The EMIM classifies firms into 240 categories. We used the series of sales of class 312111, *i*.*e*. Soft Drink Manufacturing, according to the North American Industrial Classification System (NAICS) [17]. This class includes establishments primarily engaged in manufacturing carbonated, non-carbonated drinks and other non-alcoholic beverages. Thus cola, soda, plain water, hydrating or energy beverages and juices are included in this class.

Manufacturers report sales from domestic production, which includes exports and excludes imports. Sales are reported in monetary terms (Mexican pesos) and volumes (liters). Due to confidentiality, the data are aggregated at the national level so they are unavailable by region, city or firm.

### Analysis and variables

For this study, SSB included: cola carbonates, non-cola carbonates (including sport drinks) and juices (excludes 100% juices). Some untaxed beverages are included in this broad category such as carbonates and non-carbonates with artificial sweeteners but the data do not allow us to distinguish them. The survey allowed us to distinguish plain water manufactured by the beverage industry (included in class 312111). Water manufactured by other companies dedicated to water purification and bottling (class 312112) is available but in monetary terms not in liters, therefore we did not include this category.

We first explored unadjusted trends in annual sales of SSB and plain water in liters and in liter per capita between 2007 and 2015. For descriptive statistics only, we added all sales within a year and divided by the projected population [18] to obtain liters per capita per year.

Then we ran an ordinary least squares (OLS) regression to estimate changes in SSB after the tax was implemented adapted from Chapa Cantú et al. [19]. The dependent variable is sales of SSB in liters per capita (liters/population) in a specific month, log transformed as the distribution for liters is skewed. The main variable is an indicator of the pre and post-tax period (equals 1 if 2014–2015, 0 if 2007–2013). We also explored changes in sales for the two post-tax years separately compared to the pre-tax period. We adjusted the models for seasonality using binary variables for each quarter and for changes in economic trends using the global indicator of the economic activity. The global indicator of the economic activity (GIEA) tracks short term trends in the economy [20]. This indicator incorporates weighted information on production from all sectors (primary, secondary and tertiary sectors) and reaches 93% of the gross value added. The GIEA is a short-term indicator for the Gross Domestic Product. Since the EMIM has monthly data, we preferred the GIEA that is available monthly rather than quarterly information from the Gross Domestic Product.

Results from the analyses of changes in SSB sales may be biased if the contribution of untaxed beverages included in the EMIM was large and changed significantly in the post-tax period. To assess this potential bias, we retrieved sales information from Euromonitor Mexico to evaluate the proportion of these untaxed beverages with respect to all SSB and trends over time (2007–2015) [21]. These untaxed beverages from Euromonitor included: low calorie cola carbonates, flavored and functional bottled water. We separately included sales of 100% fruit juices to look at potential substitutions.

## Results

**Fig 1:**
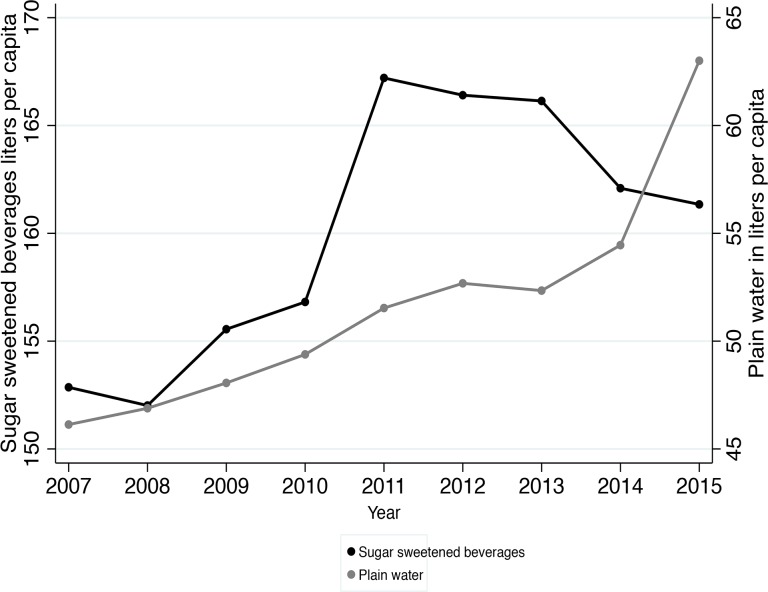
Annual sales of sugar-sweetened beverages and plain water in liters per capita, EMIM 2007–2014.

Fig 1 shows trends in annual sales of SSB and plain water in liters per capita from 2007 to 2015. The graph shows an increasing trend in SSB sales from 2007 to 2011 followed by a slight decline that became steeper in 2014. Plain water shows an increasing trend since 2007 that accelerated since 2014.

Table 1 shows unadjusted sales of SSB and plain water in liters and liters per capita. The relative difference in unadjusted sales in liters of SSBs in 2014 (the year the tax was implemented) compared to the previous pre-tax period (2007–2013) is +6.4% and in 2015 it was +7.0%. When annual sales are compared taking population size into account (dividing sales in liters by total population for each year), the difference in 2014 is less then when a comparison is performed using liters: +1.6% in 2014 and +1.1% in 2015, both compared to the pre-tax period of 2007–2013.

**Table 1:** Sales of sugar-sweetened beverages and water in liters and liters per capita in the pre and post-tax period, EMIM 2007–2014.

Sales and period	Sugar-sweetened beverages	Plain water
**Sales in liters (millions)**		
Pre-tax 2007–2013	18,237	5,667
Post-tax 2014	19,404	6,518
Post-tax 2015	19,523	7,623
Relative difference 2014 compared to 2007–2013	6.4%	15.0%
Relative difference 2014 compared to 2007–2013	7.0%	34.5%
**Sales in liters per capita**		
Pre-tax 2007–2013	159.5	49.6
Post-tax 2014	162.0	54.4
Post-tax 2015	161.3	63.0
Relative difference 2014 compared to 2007–2013	1.6%	9.8%
Relative difference 2015 compared to 2007–2013	1.1%	27.1%

Source: Authors’ estimations using the EMIM 2007–2015.

Table 2 shows the results of the models estimating changes in SSB and plain water sales before and after the tax was implemented adjusting for the global indicator of economic activity and seasonality. Model 1 for SSB shows a decline in sales of 7.3% for the 2 years post-tax (2014–2015) compared to the pre-tax period (2007–2013). Model 2 shows a decline of 6.2% in 2014 and 8.7% in 2015 compared to the pre-tax period. For plain water, model 1 shows an increase of 5.2% in sales (2014–2015) whereas model 2 shows a significant increase of 11.8% in 2015 only. In all models, the global indicator of the economic activity was highly correlated with sales.

**Table 2:** Changes in sales of sugar-sweetened beverages and plain water (liters/capita) after the tax was implemented, EMIM 2007–2014.

Variable	Sugar-sweetened beverages		Plain water	
	Model 1	Model 2	Model 1	Model 2
2 year post-tax period ^a^	-0.073**	-	0.052*	-
Post-tax year 2014 ^a^	-	-0.062**		0.002
Post-tax year 2015 ^a^	-	-0.087**		0.118**
**Quarter** ^**c**^				
Quarter 2	0.134**	0.134**	0.190**	0.191**
Quarter 3	0.110**	0.110**	0.137**	0.139**
Quarter 4	0.023	0.026+	-0.025	0.020
Logarithm of the global indicator of the economic activity	0.760**	0.833**	1.088**	1.013**
R squared	0.68	0.68	0.74	0.78
Observations	108			

Source: Authors’ estimations using the EMIM 2007–2015. Ordinary Least Square Regression.

** Significant at 1%

* significant at 5%, + significant at 10%

^a^ reference group pre-tax period 2007–2013.

^c^ Binary variables for each quarter of the year to adjust for seasonality.

As shown in Table 3, data from Euromonitor Mexico shows that low calorie soda carbonates increased from 21.6 in 2007 to 27.9 ml/capita/day in 2013 but there was no increase in these beverages after the taxes were implemented. Low calorie beverages represent less than 6% of total beverages (excluding bottled water and 100% juices). Table 3 also shows increases in flavored and functional bottled water over the entire period. Although this category includes beverages with added sugar and sugar free drinks preventing us to separate taxed and untaxed beverages, the data show that the contribution to total beverages excluding water increased slightly in the post-tax period. 100% juices represent a very small share and have not increased significantly over time.

**Table 3:** Trends in low calorie carbonates, flavored water and 100% juices in Mexico, 2007–2015 (ml/capita/day).

Type of beverage	2007	2008	2009	2010	2011	2012	2013	2014	2015
Low calorie cola carbonates	21.6	22.7	24.1	25.5	26.3	27.1	27.9	27.7	27.7
Flavored and functional bottled water*	28.8	27.7	29.0	32.3	34.0	34.8	35.9	36.4	37.3
% Low calorie cola carbonates/ all beverages excluding water	4.6%	4.8%	5.1%	5.4%	5.4%	5.5%	5.6%	5.8%	5.8%
% Low calorie cola carbonates and flavored/functional water/ all beverages excluding water	10.6%	10.7%	11.2%	12.2%	12.4%	12.5%	12.9%	13.4%	13.6%
100% fruit juice	4.9	5.2	4.9	5.2	5.5	5.8	5.8	6.0	6.0

Source of data: Euromonitor.

*Flavored bottled water includes both carbonates and still, the product can be either sugared or sugar-free. Functional bottled water includes water that has been altered to include vitamins, minerals, fruits or herb.

## Discussion

We used the EMIM monthly series to estimate changes in sales of SSB and plain water before and after the tax was implemented. For SSBs, comparing unadjusted sales in millions of liters would lead to conclude that sales increased after the tax implementation. By only taking into account the population, results show much smaller increases. In contrast, when using a statistical model that adjusts for seasonality and economic activity, results showed a 7.3% sales reduction of SSB per capita in the 2-year post-tax period and reductions of 6.2% in 2014 and 8.7% in 2015 compared to the pre-tax period. The model showed the importance of adjusting for the GIEA. The rational is as follows: increased economic activity reflects an overall growth in the domestic production and as a consequence, there could be more resources available in the country to spend. Two potential effects are plausible: either the supply of beverages increased or the demand for beverages raise as a result of these additional resources available. In both cases, sales may increase with economic activity independent of the tax. The analyses presented in the study allow estimating changes in sales between periods that had different economic growth rates. For water, in contrast, the unadjusted data overestimated the increase in sales, while the model shows a 5.2% increase in plain water sales (manufactured by the non-alcoholic beverage industry) compared to the pre-tax period (2007–2013).

The 6.2% decline in SSB sales in 2014 documented in this study is close to our previous study documenting a 6% decline in household purchases of taxed beverages in which consumer panel data were used [14]. In contrast, this study did not find an increase in plain water for 2014 as did our previous study, which estimated an increase of 4%. The differences may be related to the category included in this study (water manufactured by the beverage industry) as water from purifying and bottling companies was excluded.

The study has limitations. The first limitation is that the SSB category includes beverages with artificial sweeteners added (untaxed beverages). Using information from Euromonitor we showed the small contribution of these beverages no significant increases (particularly for low-calorie sodas) in the post-tax period. Therefore, changes in SSB beverages documented in this study do not seem to be biased by the inclusion of untaxed beverages. 100% fruit juices represent a very small proportion of beverages in the country and sales have not increased after 2014 suggesting no substitution for beverages with calories.

Secondly, our source of information includes exports that have no taxes and excludes imports. Nevertheless, this does not represent a concern, since according to the United Nations Comtrade database [22], the commercial balance of Mexico regarding SSB is of 0.63% of the apparent national SSB consumption. We did not include the commercial balance in the analysis because it is available on a monthly basis only since 2011.

The third limitation is that we could only include plain water manufactured by the non-alcoholic beverage industry. According to Euromonitor data, sales of plain water reached 161 liters per capita in 2014 whereas the EMIM shows only 54.4 liters per capita for class 312111.

A fourth limitation is that we were not able to adjust for other variables that may affect sales independent of the tax. For instance, temperatures were particularly high in 2015 relative to previous years. Also, the soft drinks manufacturing industry could have increased expenditures in advertisings and promotion of their products. In contrast, the implementation of the tax on SSB received media coverage, which could have increased awareness about possible health negative consequences of the consumption of SSB. However, although we lack of information on these factors, the binary variable for the pre and post-tax period captures all other interventions/strategies that happened in each period. If promotions and publicity were larger than any other strategy to strengthen the fiscal policy in the country, the current results maybe downward biased. If industry strategies had a positive effect on sales in the post-tax period, the real effect of the tax may be higher, in absence of any other relevant intervention in favor of the tax.

Lastly, we acknowledge that in the absence of an experimental design, given that the tax was implemented in all regions in the country, causality cannot be claimed. However, the analysis adjusts for factors that change with time independent of taxes and are associated with sales: population and economic activity.

The strength of this study is that it relies on aggregated sales data that is an adequate proxy for consumption as underreporting is less likely compared to household or individual level self-reported data. However, the use of household panel data, such as the data used in the previous study that documented a 6% reduction in purchases of taxed beverages, is superior for evaluating these types of policies since it allows a better classification of taxed and untaxed beverages, finer adjustments of influencing factors at the household level as well as a larger sample size.

This paper illustrates the relevance of considering population growth (presenting sales per capita) and adjusting statistically for variables that change over time and that are associated with the demand for beverages when comparing sales over time for assessing effects of policies such as the SSB tax in Mexico. The use of unadjusted aggregate sales is clearly inappropriate.

The findings from this study reiterate results from our previous publication using beverage purchases from a household panel indicating effects of the tax in reducing purchases of taxed beverages and increasing purchases of untaxed beverages during the first year of implementation of the tax in Mexico. Results of this study provide additional evidence indicating the effectiveness of the tax on SSB in reducing sales of SSB and increasing sales of plain water both in the first (2014) and second year (2015) after the implementation of the tax.

An average 7.3% decline in SSB consumption after two years of implementation of the tax could have an impact on health. As the tax is modest, greater impacts on consumption may be achieved if the tax was increased. Further research should be conducted to monitor changes in sales and consumption in the long terms and to evaluate impacts on health outcomes.
